# Dose Reduction to the Scalp with Hippocampal Sparing Is Achievable with Intensity Modulated Radiotherapy

**DOI:** 10.4236/ijmpcero.2014.33023

**Published:** 2014-08

**Authors:** Matthew Witek, Yelena Vahknenko, Joshua Siglin, Amy Harrison, Ying Xiao, Haison Lui, David Andrews, Wenyin Shi

**Affiliations:** 1 Department of Radiation Oncology, Thomas Jefferson University Hospital, Philadelphia, USA; 2 Department of Neurosurgery, Thomas Jefferson University Hospital, Philadelphia, USA

**Keywords:** Hippocampal, Scalp Sparing WBRT, IMRT

## Abstract

We evaluated the feasibility of combined hippocampal- and scalp-sparing intensity-modulated radiotherapy (IMRT) plans. This study included 7 patients who received conventional palliative whole brain radiation treatment (WBRT) for brain metastasis. The brain, hippocampus, and scalp were contoured and replanned with intensity modulated radiation therapy. The prescription dose was 30 Gray (Gy) in 10 fractions with hippocampus and normal structure constraints per the Radiation Therapy Oncology Group (RTOG) 0933 protocol. Further planning was done to minimize the scalp dose while maintaining the dose constraints for the hippocampus. Dose volume histograms were obtained from conventional opposed lateral fields, IMRT and compared. Planning target volume (PTV) coverage for all plans fell within the RTOG 0933 critical structure acceptable variation category. When compared to traditional opposed lateral fields, the IMRT plan with combined hippocampal- and scalp-sparing constraints was able to significantly reduce the max and mean scalp dose as well as the percentage of scalp receiving 10 and 20 Gy by 46% and 35%, respectively, while maintaining acceptable RTOG 0933 hippocampal dose variations. We conclude that acceptable PTV coverage and sparing of the scalp and hippocampus can be accomplished using a 9-field non-coplanar IMRT plan. Prospective study is warranted to understand the impact on radiation induced alopecia.

## 1. Introduction

Brain metastases account for approximately 15% - 30% of all intracranial tumors, and it is estimated that 25% - 30% of patients with cancer and 50% of patients dying from their disease will develop brain metastases [1]. These lesions frequently cause debilitating symptoms that have a significantly negative impact on the patient’s health-related quality of life. In addition, the presence of brain metastases portends a poor prognosis with median survival ranging from 1 month without treatment to 18 months for patients with favorable prognostic factors undergoing a multidisciplinary treatment approach typically including surgery, radiation therapy, and medical management [2]. Given the palliative nature of these treatment modalities, recent efforts have focused on devising techniques that limit their side effect profile without sacrificing local tumor control that best allows for the maintaining of a meaningful quality of life.

WBRT, a mainstay in the treatment of brain metastases, has been shown to improve the 1 – 2 month median survival of patients with brain metastases treated with corticosteroids to approximately 6 months [3–5]. This benefit is further improved when WBRT is either preceded by surgical resection or followed by a radiosurgery boost [6–8] Notwithstanding these benefits, WBRT is recognized to cause early neurocognitive decline within the first 1 – 4 months following treatment that mainly affects memory whereas in the long-term, effects include cognitive deterioration in domains other than memory and cerebellar dysfunction [9]. The preponderance of data suggests that the pathogenesis of WBRT associated neurocognitive deficits involves radiation injury to the sub- granular zone of the hippocampi that contains proliferating neuronal progenitor cells [10] [11]. In light of these findings, the RTOG conducted a phase II trial (RTOG 0933) testing the hypothesis that hippocampal-sparing WBRT delivered to patients with brain metastases may delay or reduce the onset, frequency, and/or severity of neurocognitive decline. Indeed, analysis of these data demonstrated that hippocampal sparing WBRT reduced neurocognitive deficits, as mean relative decline in the Hopkins Verbal Learning Test-Delayed Recall from baseline to 4 months was 7.0%, which was significant in comparison to the historical control (p = 0.0003). This was achieved with less than 5% of patients progressing in the hippocampal avoidance area [12]. Given these results, further evaluation of this technique is being applied in a phase two trial in patients with small cell lung cancer receiving prophylactic cranial irradiation (NCT01797159).

In addition to neurologic side effects, WBRT causes distressing physical side effects, most notably iatrogenic alopecia. In a study by Irvine *et al.,* patients reported that along with fatigue, hair loss was the most distressing physical symptom associated with the WBRT [13]. In an attempt to attenuate this symptom, the following study evaluated the feasibility of treating the whole brain while meeting current RTOG 0933 hippocampal dose requirements and employing a scalp constraint that may reduce or eliminate radiation-induced alopecia.

## 2. Material and Methods

### 2.1. Patient Selection

Seven patients who had previously undergone CT simulation of the brain for conventional WBRT between June 2011 and December 2011 at Thomas Jefferson University Hospital for brain metastases were selected for repeat planning. Patients with metastases within 5 mm of either hippocampus were excluded. The patients had previously undergone non-contrast computed tomography (CT) scan with aquaplast mask fixation. Additionally, all patients had previously received MRI of the brain including T1 post-contrast, T2, and fluid attenuation inversion recovery (FLAIR) sequences. These scans were fused to the CT simulation images and contouring was subsequently performed using Xio FocalSim (Elekta North America, Norcross, GA). The hippocampus was contoured as previously described by Gondi *et al.* [14]. Additionally, normal tissue structures including the optic nerve, optic chiasm, brainstem and whole brain were contoured. Scalp contours were created using a 5 mm expansion from the skull surface. Institutional review board approved anonymized patient data for planning research.

#### 2.1.1. Opposed Lateral Planning

Standard opposed lateral fields using 6 MV photons were planned in Xio^®^ planning software (Elekta, Stockholm, Sweden) to cover the cranial contents with a 1 cm margin on the base-of-skull and extending into air around the cranial vault. The collimator was angled to follow the base-of-skull and the inferior border was blocked at the C1 and C2 vertebral body disk space. The chosen prescription point was mid-plane in the center of the clinical target volume (CTV). Cumulative dose-volume histograms were then produced for the PTV, hippocampi, and scalp.

#### 2.1.2. IMRT Planning

Patients were simulated in the supine position with their arms down. CT images (GE Light Speed RT 16 CT Scanner, Providian Medical Equipment LLC., Willowick, Ohio) for 30 degrees head holder angle were artificially generated by rotating CT scans derived with 0 degree position. Original CT scan was imported to MIM vista software system (version 5.1.2; MIMvista Corp., Cleveland, OH), rotated along left-right axis (pitch) by 30 degrees, and then re-sampled to create a new CT image that mimics a 30 degrees head angle position. The contours in the 0 degree CT image were then transferred to the rotated image by fusing two images together through rigid registration.

Inverse-planning was performed using Xio^®^ planning software (Elekta) and 6 MV photons. Clinical criteria and inverse planning algorithm constraints for the LINAC-based IMRT planning are listed in **Table 1.** The same data set previously used for the conventional plan was transferred to the inverse planning system. The number and directions of beams were chosen empirically in order to optimize scalp sparing and PTV dose conformality. The chosen geometry had a total of 9 non-coplanar beam angles. Optimization parameters were chosen in an attempt to obtain a homogenous coverage of the PTV and to minimize the dose to the scalp. The dose was scaled to cover 90% of the PTV to meet RTOG 0933 constraints.

#### 2.1.3. Statistical Analysis

Comparisons of dose volume histogram parameters were assumed to be normally distributed and were analyzed with the Student’s paired *t*-Test with an upper bound of p < 0.05.

## 3. Results

Acceptable PTV coverage is achievable with combined hippocampal- and scalp-sparing IMRT. We evaluated whole brain PTV coverage of 7 patients planned with opposed laterals, hippocampal-sparing IMRT, and combined hippocampal- and scalp-sparing IMRT plans by comparing values generated by their respective dose volume histograms. According to RTOG 0933, 90% of the PTV is required to be covered by the prescription dose of 30 Gy with 2% of the PTV receiving no more than 37.5 Gy and at least 98% receiving 25 Gy. Despite better PTV coverage and lower maximum point doses with the opposed lateral field arrangement, both hippocampal- sparing and combined hippocampal- and scalp-sparing IMRT met the RTOG 0933 constraints **(Table 2).**

**Figure 1:**
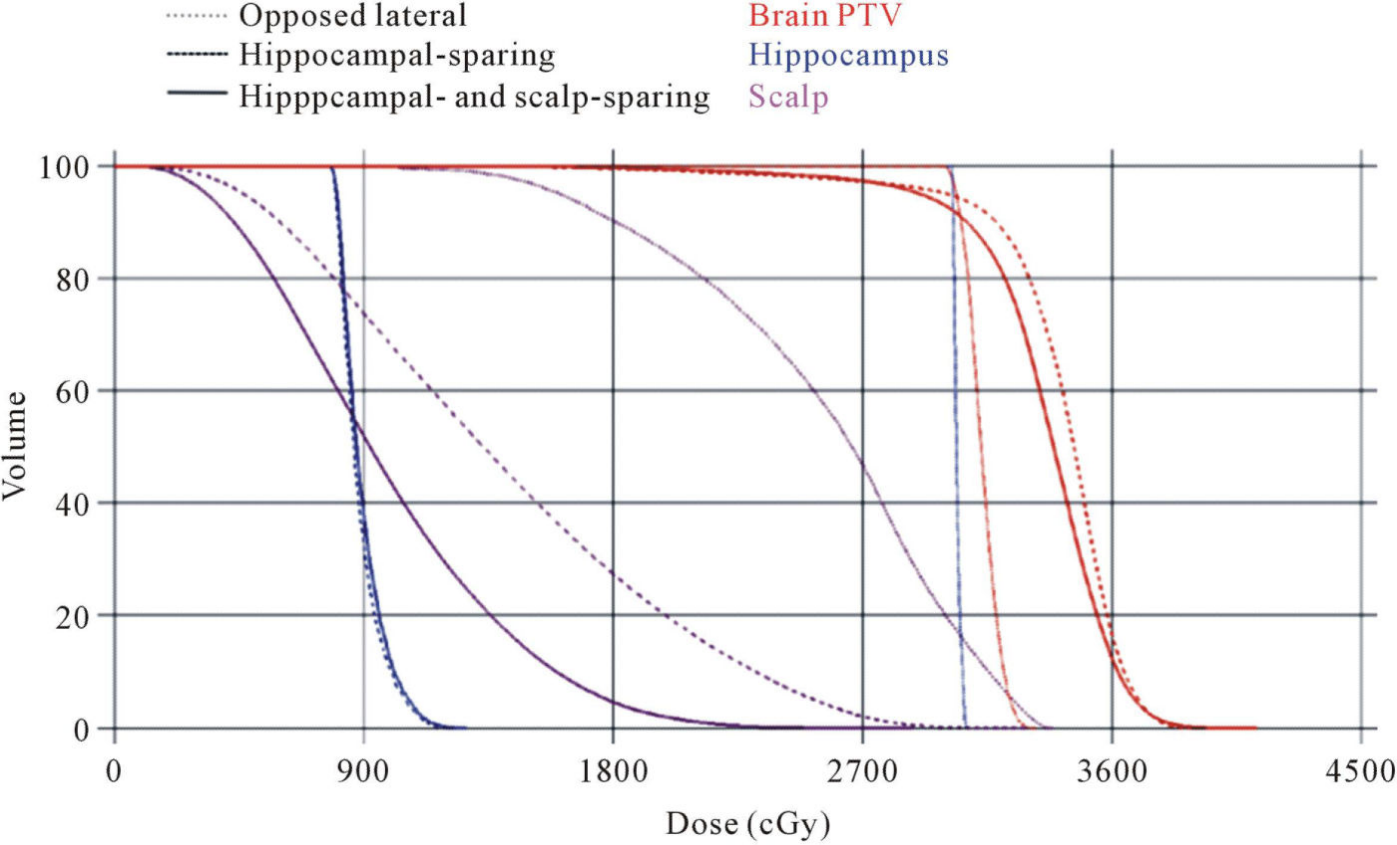
DVH for oppposed lateral, hippocampal-sparing, and hippocampal- and scalp-sparing IMRT plans. There was acceptable PTV coverage for all three plans. There was significant improvement in scalp doses with the hair-sparing IMRT plan. This reduction in scalp dose was maintained when hippocampal-sparing constraints were employed.

Scalp dose reduction with acceptable hippocampal-sparing and PTV coverage is feasible. Having already demonstrated acceptable PTV coverage with a combined IMRT plan employing hippocampal and scalp constraints, we next evaluated the feasibility of meeting hippocampal-sparing goals while reducing the dose to the scalp. Dose parameters of the hippocampus and scalp were compared between all three plans **(Figure 1).** We noted no significant difference in maximum, minimum, and mean hippocampal doses between the hippocampal- sparing (maximum: 14.5 ± 1.1 Gy; mean 9.2 ± 0.6 Gy; minimum 7.5 ± 0.3 Gy) and combined hippocampal- and scalp-sparing (maximum: 15.1 ± 0.8 Gy; mean 10.0 ± 0.9 Gy; minimum 8.1 ± 0.9 Gy) (maximum: p < 0.3; mean p < 0.1; minimum p < 0.12) for IMRT plans. Both IMRT plans met the acceptable variations dose requirements of RTOG 0933 (Table 3). We next assessed scalp maximum and mean values, and scalp volumes receiving at least 10 and 20 Gy between the opposed lateral plan and the 9-field combined hippocampal- and scalp-sparing IMRT plan and noted significant improvement in every parameter. For the opposed lateral plan the values were: maximum: 31.2 ± 0.4 Gy; mean 21.8 ± 1.1 Gy; V20 Gy: 68.0% ± 5.7%; V10 Gy: 96.7% ± 3.5% and for the scalp-sparing plan: maximum: 29.2 ± 1.9 Gy, p < 0.001; mean 9.8 ± 0.6 Gy, p < 0.001; V20 Gy: 2.4% ± 1.1%, p < 0.001; V10 Gy: 45.1% ± 4.9%, p < 0.001. To ensure that the improved scalp doses seen by the addition of a scalp constraint was responsible for the reductions noted above and was not a reflection of the hippocampal- sparing constraints or IMRT technique, we compared scalp dose parameters between the scalp constraint containing and non-scalp constraint containing plans (maximum: 34.8 ± 1.6 Gy; mean 15.4 ± 1.5 Gy; V20 Gy: 28.8% ± 7.2%; V10 Gy: 74.4% ± 6.3%) and noted significant improvement in all parameters in the plan with the scalp constraint (Table 3).

**Figure 2:**
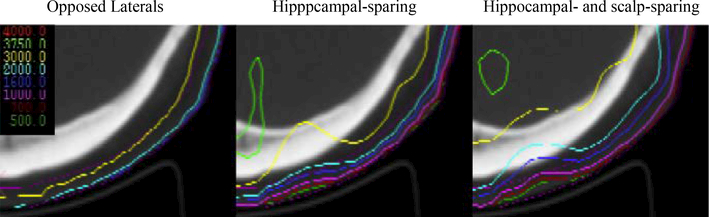
DVH for oppposed lateral, hippocampal-sparing, and hippocampal- and scalp-sparing IMRT plans. Isodose lines between all three plans revealed a noticeable difference between isodose lines less than 30 Gy. Isodose lines less than 30 Gy are almost completely on the surface in the opposed lateral plan where as in the IMRT plan, there is deepening of the 10, 16, and 20 Gy isodose lines.

A comparison of isodose lines between opposed lateral and combined hippocampal- and scalp-sparing IMRT plans revealed a noticeable difference between isodose lines less than 30 Gy (Figure 2). In both axial and sagittal plans, isodose lines less than 30 Gy are almost completely on the surface in the opposed lateral plan where as in the IMRT plan, there is deepening of the 10, 16, and 20 Gy isodose lines. Also noted, the IMRT plan provides almost complete coverage of the PTV by the 32 Gy isodose line re-enforcing the overall increase in dose of this plan. Visual review of the IMRT plans revealed that the maximum point doses were in the PTV.

## 4. Discussion

Here, we demonstrate the feasibility of combined hippocampal- and scalp-sparing WBRT using a 9-field IMRT plan and incorporating a 30 degrees head angle. The scalp-sparing IMRT plan allowed for a significant reduction in the scalp maximum, mean, V10 and V20 values when compared to a conventional opposed lateral WBRT plan. The improved scalp doses were the result of the scalp constraint, as a similar IMRT plan sparing only the hippocampus did not generate the same favorable scalp dosimetry. More importantly, acceptable PTV coverage was maintained when both hippocampal and combined hippocampal- and scalp-sparing IMRT planning was performed.

Temporary alopecia is a dose-dependent phenomenon that occurs at approximately 2 – 3 weeks after initiation of radiotherapy and typically resolves within 2 – 3 months after WBRT [15]. Historical data from survivors of the atomic bomb in Hiroshima suggest epilation can occur at doses as low as 0.75 Gy, while doses as low as 2 Gy in a single fraction of external beam RT have been shown to cause temporary alopecia [16–18] In contradistinction to low doses associated with temporary alopecia, higher doses of RT such as 36 Gy in 2 Gy fractions can result in permanent hair loss in 0% - 80% of patients [19] Thus, for the dose of radiation therapy received during WBRT, one could expect temporary alopecia. At a mean scalp dose of 10 Gy with scalp-sparing IMRT, the achieved 55% reduction in dose is above the apparent threshold of 2 Gy for temporary alopecia and would likely not prevent temporary alopecia. However, given that the severity of alopecia appears to be related to the total dose, our improvements may decrease the amount of hair loss [20] Indeed the level of alopecia at 10 Gy can be estimated from previous data on X-ray epilation for the treatment of tinea capitis. The radiation dose from this technique ranged from 5 Gy to 8 Gy and resulted in generalized alopecia in 20% of the treated patients, which is on the low end of the reported 80% risk of alopecia from conventional 36 Gy of cranial radiation therapy [21] This improvement in severity and duration may be enough to improve the reported distress patients note from radiotherapy-induced alopecia.

Roberge *et al.* previously described the scalp sparing benefits of IMRT [22]. In their experience, the utilization of IMRT was able to reduce the average measured dose by 53% from 95% of the prescription dose with the conventional plan to 44% with the IMRT plan. Here, we similarly were able to reduce the calculated mean scalp dose by approximately 45%. Moreover, in this study, scalp dose reduction was done in conjunction with meeting RTOG 0933 acceptable deviation dose parameters for hippocampal-sparing. As noted in the above study, performing such complex planning for a palliative treatment burdens the healthcare system. However, given our findings, these concerns are unwarranted as the possible side effect reduction and resulting improvement in quality of life can be performed in the context of hippocampal-sparing IMRT, which is currently being tested in a national clinical trial. Thus if hippocampal-sparing proves beneficial, it is not unreasonable to evaluate the impact of a scalp constraint in a future clinical trial in combination with hippocampal-sparing.

One limitation of the IMRT technique chosen is in the complexity of planning as well as time on the table. RapidArc and volume modulated radiation therapy (VMAT) techniques are gaining momentum in the treatment of many sites given their quick delivery times. In our initial experience with these planning techniques, similar scalp dosimetry was achievable; however, dual sparing of the hippocampus and scalp was not, which may limit clinical utility of arc delivery.

## 5. Conclusion

We demonstrate here for the first time the feasibility of combined hippocampal- and scalp-sparing IMRT. Reductions to the average scalp dose by approximately 45% while maintaining adequate PTV coverage and hippocampal-sparing are achievable. The clinical implications of such a technique could be addressed in future trials evaluating hippocampal-sparing as we show no change in dose reduction to the hippocampi or for PTV coverage.

## Figures and Tables

**Table 1:** Clinical criteria and inverse planning algorithm for planning.

Structure	IMRT Dose Goals
Whole brain PTV	Max Dose: 110% of prescription dose Min Dose: 95% of PTV covered by 30 Gy
Hippocampus	Max Dose: 16 Gy; D100% < 9 GY
Lenses	More table copy^a^

**Table 2:** RTOG 0933 PTV.

Per Protocol	Acceptable Deviations	Deviations Unacceptable
D2% ≤ 37.5 Gy;	D2% > 37.5 Gy. D2% ≤ 40 Gy.	V30 < 90%;
D98° b ≥ 25 Gy	D98 < 25 Gy	D2% > 40 Gy

Abbreviations: Gy = Gray; PTV = planned target volume; D2% = Maximum dose to 2% of the PTV; D98% = Maximum dose to 98% of the PTV; V30 = Volume of PTV receiving ≥ 30 Gy.

**Table 3:** Hippocampal and scalp dose parameters.

Hippocampal Sparing							
	Scalp Data				Hippocampal Data		
	
	Max	Mean	V20	V10	Max	Mean	Min
Mean	34.8	15.4	28.8	74.4	14.5	9.2	7.5
S.D.	1.6	1.5	7.2	6.3	1.1	0.6	0.3
Hippocampal and Scalp-Sparing							
	Scalp Data				Hippocampal Data		
	
	Max	Mean	V20	V10	Max	Mean	Min
Mean	29.3	9.8	24	45.1	15.1	10.0	8.1
S.D.	1.9	0.6	1.1	4.9	0.8	0.9	0.9
Opposed Laterals							
	Scalp Data						
	Max		Mean		V20	V10	
Mean	31.2		21.8		68	96.7	
S.D.	0.4		1.1		5.7	3.5	

Abbreviations: Gy = Gray; PTV = planned target volume; D100% = Maximum dose to 100% of the PTV; V20 = Volume of PTV receiving ≥ 20 Gy; VI0 = Volume of PTV receiving ≥10 Gy; IMRT = intensity-modulated radiotherapy; Max = maximum; Min = minimum; S.D. = Standard deviation.
